# A kinetic Monte Carlo simulation method of van der Waals epitaxy for atomistic nucleation-growth processes of transition metal dichalcogenides

**DOI:** 10.1038/s41598-017-02919-2

**Published:** 2017-06-07

**Authors:** Yifan Nie, Chaoping Liang, Pil-Ryung Cha, Luigi Colombo, Robert M. Wallace, Kyeongjae Cho

**Affiliations:** 1 Department of Materials Science and Engineering, The University of Texas at Dallas, Richardson, Texas 75080 United States; 2 School of Advanced Materials, Kookmin University, Jeongneung-gil 77, Seongbuk-gu, Seoul 136-702 Korea; 3 Texas Instruments Incorporated, 13121 TI Boulevard, Dallas, Texas 75243 United States

## Abstract

Controlled growth of crystalline solids is critical for device applications, and atomistic modeling methods have been developed for bulk crystalline solids. Kinetic Monte Carlo (KMC) simulation method provides detailed atomic scale processes during a solid growth over realistic time scales, but its application to the growth modeling of van der Waals (vdW) heterostructures has not yet been developed. Specifically, the growth of single-layered transition metal dichalcogenides (TMDs) is currently facing tremendous challenges, and a detailed understanding based on KMC simulations would provide critical guidance to enable controlled growth of vdW heterostructures. In this work, a KMC simulation method is developed for the growth modeling on the vdW epitaxy of TMDs. The KMC method has introduced full material parameters for TMDs in bottom-up synthesis: metal and chalcogen adsorption/desorption/diffusion on substrate and grown TMD surface, TMD stacking sequence, chalcogen/metal ratio, flake edge diffusion and vacancy diffusion. The KMC processes result in multiple kinetic behaviors associated with various growth behaviors observed in experiments. Different phenomena observed during vdW epitaxy process are analysed in terms of complex competitions among multiple kinetic processes. The KMC method is used in the investigation and prediction of growth mechanisms, which provide qualitative suggestions to guide experimental study.

## Introduction

Since the isolation of graphene and the re-introduction of layered two-dimensional (2D) materials^1–3^ and the subsequent revival of single or few layer transition metal dichalcogenides (TMDs)^4^, this family of 2D TMD materials has attracted great research interest. They possess unique electronic and optical properties which not only show potential for new applications^5–13^, but also have become a significant material platform of several new fields in fundamental physics^14, 15^. Many studies have presented the versatility of this group of materials, and in order to make the TMDs accessible for practical applications, a more effective and scalable production technique must be developed to replace laboratory-scale mechanical exfoliation method^4, 16–18^. “Bottom-up” synthesis methods are currently being developed to achieve a controlled synthesis of TMD layers. The TMD growth methods fall within a group of new techniques called the van der Waals (vdW) epitaxy. This nomenclature comes from the fact that the vertical interaction between the growing layers as well as the substrate are weakly interacting between van der Waals gaps rather than other stronger interactions. Such weak, yet not negligible interaction, makes the process different in many subtle aspects from the traditional growth mechanisms of 3D materials (*e*.*g*., metal on metal, Ge on Si). Various epitaxy techniques have been proposed for vdW materials, including chemical vapor transport or reaction^7, 9, 19, 20^, molecular beam epitaxy (MBE)^21–23^, metal-organic chemical vapor deposition (MOCVD)^24^, *etc*. These experimental techniques have been applied and have achieved limited success in controlled growth of TMDs.

Fundamental understanding of a new material requires high quality synthesis. Specifically, a production of high-quality TMD thin films requires a control of structural properties such as grain size, phase, point defects, doping level, layer number, stoichiometry, surface roughness and more. One of the major experimental difficulties is the identification of key factors that control each attribute of the growing TMD samples. It is usually very time-consuming to establish the experimental conditions of crystal growth and it is especially difficult to make the direct connection between the experimental parameters and the kinetic growth processes in the atomic scale. These difficulties are compounded by the unusual nature of vdW epitaxy in contrast to conventional epitaxy of strongly bonded crystalline materials (*e*.*g*., bulk metal, semiconductors, and insulators), leading to an absence of theoretical framework to analyze experimental studies. In order to overcome the experimental difficulties, simulation techniques could help describe the atomic scale synthesis process of the TMDs.

Currently, the most widely used and accepted simulation approach for TMDs is the static first principles method based on the density functional theory (DFT)^25–27^. The DFT simulation is able to provide reliable predictions on the structural and electronic properties of the materials. However, these methods are limited to the study of unit atomic processes (*e*.*g*., atomic adsorption/desorption, surface diffusion, edge diffusion) in modeling TMD growth processes, but are not capable of approaching a dynamic process deviating from an equilibrium and with a macroscopic time range. Another important challenge comes from the fact that most deposition systems are open systems and require an appropriate representation of addition and subtraction of matter into and out of the system. In order to address these challenges, two kinetic methods have been adapted to simulate the dynamic evolution of 2D materials system, namely the kinetic Monte Carlo (KMC)^28, 29^ and the phase field model (PFM)^30^. KMC has been used by Girit *et al*. and Kotakoski *et al*. to investigate the edge evolution of graphene and hexagonal boron nitride, respectively^31, 32^. In a simulation of the CVD of graphene, Whitesides and Frenklach have demonstrated the capability and level of details that a KMC can provide with meticulous model construction and data extraction^33^. The phase field modeling of Meca *et al*. of graphene growth shows that good agreement can be found between the simulation and experimental observations^34^. These works have provided important insights in the growth processes often hidden in the experimental data. More specifically, the KMC simulations are able to illustrate the mechanisms at the atomic scale that are usually difficult to observe experimentally. Recently, PFM and KMC models have also been developed to study more complicated 2D material systems such as the TMDs. Artyukhov *et al*. employed a phase field model to simulate the growth and crystal twinning of the TMDs^35^. A KMC model of Govind Rajan *et al*. provided an initial generalization of the evolution of domain shape in the CVD of TMDs within the continuous flake region^36^. While promising, these simulations are often designed to address individual issues and leave many other key questions related to TMD growth unanswered. In the aforementioned KMC simulations on the growth of TMDs the substrate effects were neglected. Such simplification would limit the utility of the simulations, as the substrate not only significantly affects the electric properties of the TMDs^37–39^, but more importantly, it also influences the balance of the competition on the thermodynamic stability between the single layer and the multilayer, as shown later in this work. Moreover, from a kinetic perspective, without a proper description of the substrate, it is impossible to describe the adsorption/desorption and diffusion of adatoms, which participates in the determination of key properties of the resultant film, such as film continuity, defect level, grain size, *etc*. In addition, all of the previous simulations start with a pre-existing nucleus and thus a homogeneous nucleation of additional flakes is not included in the simulation scenario. Since the initiation of a new domain or a new layer is excluded from such simulations, the simulated monolayer growth of a single grain is a reflection of the model’s limitation. Such KMC simulations represent unrealistic growth scenarios in which the major mechanisms in actual experiments are not fully incorporated. Due to these limitations, previous simulations were not able to address multiple nucleation and growth leading to grain merging and multilayer 3D growth modes, which are common observations in TMD growth experiments. In order to provide a fundamental understanding on the growth mechanism and a guidance to experimentalists, a KMC model is required to incorporate the necessary atomic processes. For this purpose, a realistic KMC simulation model was developed to study growth processes at the atomic level of TMDs during vdW epitaxy and to provide theoretical bridge between the atomic processes and microscopic events observed in experimental studies^40^.

In this study, we present the technical details of an on-lattice diffusion based kinetic Monte Carlo model to describe the vdW nucleation and growth of the TMDs. The inputs of the KMC simulation are drawn from first principles calculations, making use of the accuracy of a static simulation and the irreplaceable capability of a kinetic one. In addition to our previous results, in order to develop a predictive modeling perspective, some of the parameters will be adjusted individually in a parametric mechanism investigation. Advanced algorithms, especially a binary-tree searching engine^41^, and a high level of details make it possible to accurately simulate and effectively describe the competing factors that cause many phenomena important to the TMD material growth processes and atomic scale mechanisms. This model brings insight to the fine control of the vdW epitaxy of the TMDs. The current KMC model provides a quantitatively accurate model for MBE growth of TMDs, and with further refinements it could be used to make both qualitative and quantitative predictions for CVD growth processes.

## Design concept: algorithm and structural model

Kinetic Monte Carlo (KMC) method is a variation of the Monte Carlo methods. In contrast to the traditional Monte Carlo methods that make use of the random number to study the equilibrium condition, a KMC method is capable of studying non-equilibrium kinetic processes^29^. In a KMC method, possible kinetic events are listed as an event catalogue, and stochastic sequence of events is randomly selected by including another random number to simulate the real time. The algorithm of the KMC method consists of the following steps:

1. Initiation: calculating the rates of all possible events *r*_*i*_ and their sum ${\sum r}_{i}$;
2. Event selection and execution: generating a random number ${\xi }_{1}\in [\mathrm{0,1})$, and the *q* th process that makes ${{\sum }_{i=1}^{q}r}_{i}\le {\xi }_{1}{\sum r}_{i} < {{\sum }_{i=1}^{q+1}r}_{i}$ is chosen to proceed;
3. Time advancing: another random number ${\xi }_{2}\in (\mathrm{0,1}]$ is chosen, and the real time is advanced by ${\rm{\Delta }}t=-\frac{\mathrm{ln}\,{\xi }_{2}}{\sum {r}_{i}}$;
4. Refreshing: re-calculating the rates of all possible events *r*_*i*_ and their sum ${\sum r}_{i}$ at the new configuration;
5. Convergence: repeating steps 2 to 4 until a kinetic steady state is reached; and
6. Data collection: repeating steps 2 to 4 and collecting the data.

In this study, the criterion for the steady state is reached when the growth rate of the domain becomes constant. Our study shows a wide range of transient periods before reaching a steady state growth. In most of the cases, a steady state can be reached after a few tens of thousands Monte Carlo steps (MCSs), but in extreme cases close to equilibrium, where the net chemical reaction rarely proceeds and most of the adatoms remain within the adsorption/desorption equilibrium, a few millions of MCSs are required for reaching a steady state.

Different from other synthesis techniques, the reactions of MBE and MOCVD are mainly proceeding at the catalytically active domain edges of the TMDs. As the physical properties of TMDs are very sensitive to the layer number, it is desirable to control the layer number during its growth, through a precise control of the lateral versus vertical growth rates. Considering this growth kinetics, MOCVD and MBE methods possess an intrinsic advantage which can facilitate controlled growth. However, to simulate such growth processes using KMC method, there are several technical difficulties to overcome in the simulation of the MOCVD and the MBE processes.

Comparing with the KMC simulations on the growth of 3D materials and those of graphene, a KMC model on the vdW epitaxy of the TMDs requires qualitatively different approaches. For the simulation of elemental materials, such as the metals, silicon, and graphene, a simple bond-counting model is often sufficiently precise, due to the simple bonding structures of the bulk materials. Alloying or substitutional impurities can be presented in these models, as long as different elements play a similar role in the modeled structure. For example, a model for the growth of Co on metal substrate can be easily transformed to simulate Co-Pt alloy growth on metal substrate^42^. So is a model of silicon for SiGe or III–V semiconductors^43^, and a model of graphene for hexagonal boron nitride^32^. The structural simplicity also makes the simulation of nucleation events straightforward as bonds can be formed in the direction parallel to the substrate surface, and two adatoms can form a dimer and cease to be mobile within the common range of temperature. In contrast, the structural complexity of the monolayer TMDs (X-M-X three atomic layers; X = chalcogen, M = metal) makes it impossible to employ the existing models of either bulk metals or graphene to precisely describe the TMDs, as the metal and the chalcogen atoms constitute the X-M-X sandwiched structure with significantly different roles. To describe such non-interchangeable roles of M and X, a new structure model is required to simulate the growth of the TMDs. The major challenges of building such a model lie in the differentiation of the elements and the atomic arrangement within the unique structure of monolayer TMDs.

The differentiation of M and X elements is particularly important for the CVD and MBE processes, because the two elements come from separate precursors. In this simulation, each lattice site can change according to three states: (1) empty (labeled 0), (2) occupied by a metal atom (labeled 1) and (3) occupied by a chalcogen (labeled 2). The differentiation of the elements also increases the level of details of the simulation, because it doubles the input parameters required in the simulation.

**Figure 1:**
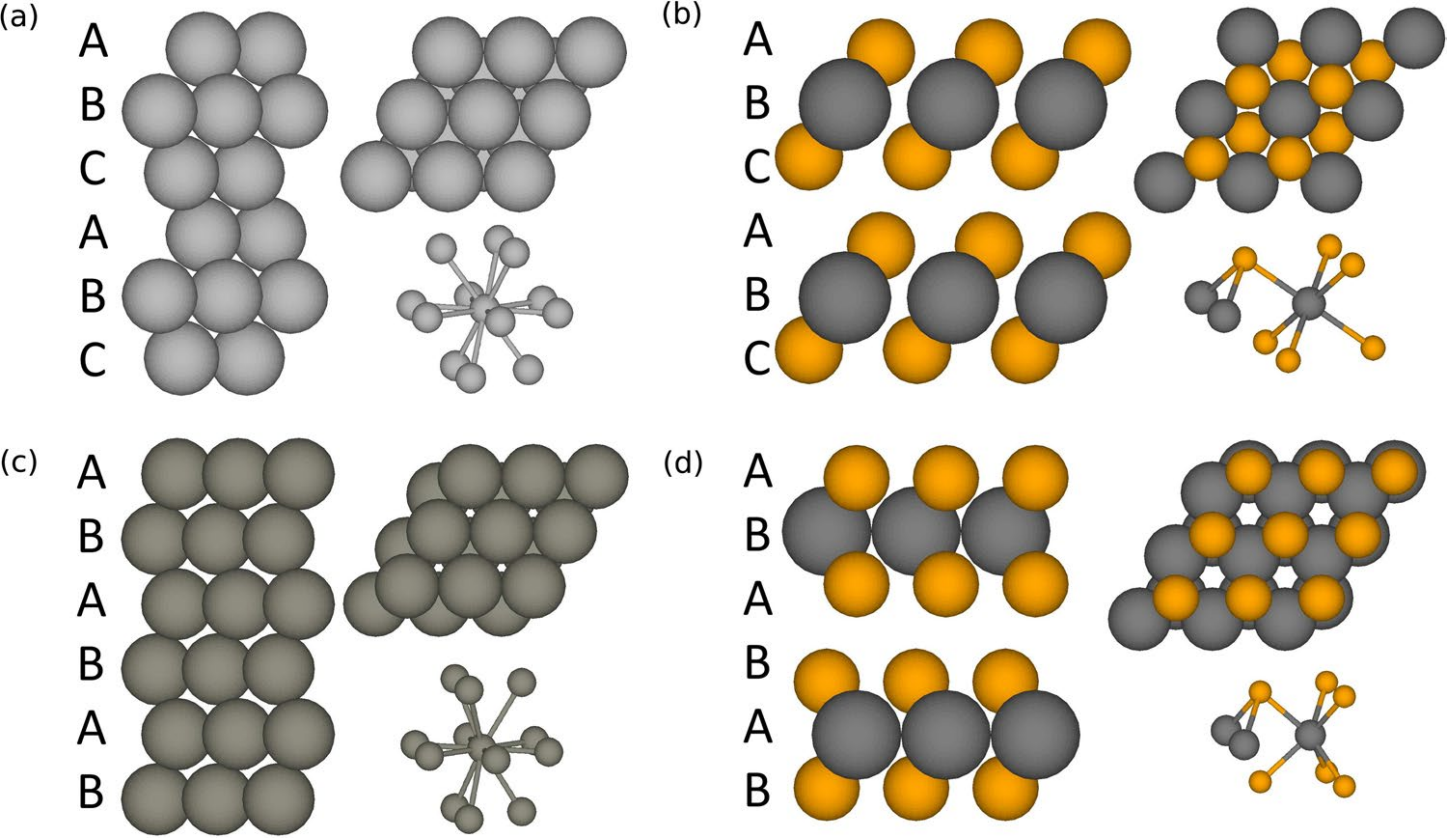
Side view (left), top view (up-right) and the neighboring environment (right-bottom) of (**a**) an fcc metal, (**b**) a 1T-TMD, (**c**) an hcp metal and (**d**) a 2H-TMD. The figure shows that the stacking of the atomic layers in a TMD resembles that in a closest packing metal, but different in the sequence of the elements and the interlayer and interatomic distances.

Being layered materials, the TMDs are frequently juxtaposed in discussion with graphene, however, the stacking sequence of the atomic layers in TMDs resembles closest packed metals (face-centered cubic (fcc) structure for 1T-TMDs, and hexagonal close packed (hcp) structure for 2H-TMDs, as shown in Fig. 1). Based on the similarity in the various structures, a model simulating the deposition of TMDs can be derived from simulation models for metals, with two major modifications: (1) there are two types of elements in the stack, metal (M) and chalcogen (X), following the stacking sequence of X-M-X-X-M-X and so forth with a vdW gap at X-X stacking; (2) different from the closest packed system, in which each atom has 12 closest neighbors (Fig. 1a,c), a metal atom in a TMD has only 6 nearest neighbors, being 3 above and 3 below, and a chalcogen atom has only 3 (Fig. 1b,d). All of the first neighbors of an atom are located out of the plane parallel to the epitaxial surface.

Different diffusion paths influence different aspects of the deposition process. The participant, locale and condition of the different diffusion paths included in the model are listed in Table 1. Adatom diffusion on a substrate influences the domain’s growth rate and the homogeneous nucleation density and it competes with edge diffusion which affects the shape and continuity of the domain. Vacancy diffusion, on the other hand, shows how fast the imperfections created during the kinetic growth processes can be annihilated during thermal relaxation. All these criteria are of crucial importance in evaluating the quality of an epitaxy method and the simulated material structure it produces, making diffusion indispensable in the simulation of the TMD synthesis.

**Table 1:** The participant, locale and condition of the different diffusion paths included in this work.

Path	Participant	Locale	Condition
Substrate diffusion	Adatom	Substrate or grown domain surface	No lateral bonds, physically or chemically bonded with the substrate below
Edge diffusion	Bonded atoms	Domain edge	Laterally bonded and incorporated into the domain
Vacancy diffusion	Vacancies (bonded atoms surrounding the vacancies)	Within domain	Laterally bonded and reside in the bulk of the domain

Both edge diffusion and vacancy diffusion within a flake belong to a generalized in-flake diffusion, as they share the common bonding characteristics within a TMD flake. They differ from each other by the local bonding configurations (Table 1). As a generalized approach, in the following examples of this work, their energy barriers are unified as the in-flake diffusion barriers. It is noteworthy that a detailed study on the migration of the vacancies should not only differentiate the energy barriers, but also include the complicated behaviors of the vacancies in a specific TMD^44–46^. Detailed studies will be carried out thoroughly with these details for a future publication.

In this model, each occupied site is available to diffusion realized by discrete atom hopping, with a few straightforward constraints: (1) the target site can only be one of the 6 in-plane neighboring sites or the 6 out-of-plane neighboring sites; (2) the target site must be empty and supported by at least one atom; (3) the atom leaving its original site should not leave any atom unsupported. Each available hopping event has its corresponding rate, which will be discussed in the following sections.

## Simulation results

The kinetic statistics and the morphology reveal complex competitions during the deposition process. By identifying the connection between the external parameters and their underlying causes in competitive reactions, one can gain a fundamental theoretical understanding of 2D vdW epitaxy, which in turn can aid to the fine tuning of the experimental conditions to achieve optimal results. In this study, MBE growth of WSe_2_ on graphene is used as a starting example (input parameter details are given in our previous publication^40^). Based on this initial example, we have performed parametric studies of the TMD growth process as a function of materials parameters. To simplify the MX_2_ system for a parametric study, the adsorption energy and the diffusion barrier on the substrate are set equal for M and X.

The level of details in this model has made it possible to trace the entire nucleation-growth process of the TMDs, including many phenomena which were unable to be simulated previously, such as homogeneous nucleation, compact-fractal transition, multilayer growth, *etc*. These phenomena often bear the physical and engineering significance. In the following, after a discussion of the parameters involved in the model, these important phenomena are briefly analyzed, with attempts to compare them with the competing microscopic processes.

Due to the limitation of the computation capability, the scale of the simulation is not able to match with the actual domain size (≥*μm* scale with ~10^7^ atoms) observed in the experiments. However, in analyzing the domain shape, the simulation size does not play an important role. Under a given growth condition, after the system reaches a steady state growth, the shape of the domain remains the same. When the adsorption rate is defined in the unit of monolayers per second and with the continuous boundary condition, the “size” of the domain is more appropriately interpreted by the coverage. When dealing with nucleation density, on the other hand, the simulation system size becomes important as it sets the “detection limit” of the nuclei density. Based on this consideration, for the growth rate and morphology study, a relatively small simulation with 100 × 100 × 7 sites are used, and for nucleation study, a larger simulation with 1,000 × 1,000 × 7 sites are implemented.

## Parameters

The results of TMD growth processes are subject to the competition of different kinetic events. According to the transition state theory, the rate of a reaction is given by

1 $$r={\nu }_{0}{e}^{-\frac{{\rm{\Delta }}{G}^{\ne }}{{k}_{B}T}},$$

where *v*_0_ is the pre-exponential factor, reflecting the thermal vibration frequency of the system, with an order of magnitude of 10^13^ s^−1^ under the experimental conditions. Δ*G* ^*≠*^, *k*_*B*_ and *T* are the activation energy, the Boltzmann constant and the temperature, respectively. While parameters like temperature have a direct connection to the experimental condition, the others like the activation energy are determined by the properties of the specific materials. Thus, the parameters in the simulation can be categorized into two groups, namely the extrinsic and intrinsic parameters. The extrinsic parameters include the macroscopic conditions that can be measured and controlled experimentally. The intrinsic parameters, on the contrary, remain fixed once the synthesis strategy is determined, because they reflect the microscopic structural and kinetic constants of the species involved in the reaction, such as the TMD in question, the precursors and reactions thereof, the substrate, *etc*. The intrinsic parameters mainly influence Δ*G* ^*≠*^ in Equation (1), while the other terms can be controlled via the extrinsic parameters.

## Extrinsic parameters

### Temperature

Temperature appears explicitly in Equation (1), hence it affects the reaction rates directly. It is noteworthy that in the CVD and MBE processes, reaction and diffusion occur on the substrate surface, therefore the temperature here refers to that of the substrate.

### Adsorption rate (flux)

The starting point of the simulation is the arrival of atoms onto the substrate. Different from the diffusion processes that are governed by Equation (1), the adsorption rate of atoms is set to a constant prior to the simulation, in the unit of monolayers per second (ML/s), as an experimentally observable parameter. The adsorption rate or the flux of adatoms can be converted from that of the precursors based on the experimental, theoretical or empirical relations^36^.

### Chalcogen to metal (C/M) ratio of the precursors

The ratio of the precursors has been reported to be an important factor due to its influence on the growth morphology and stoichiometry. In many cases, the influence of the C/M ratio is more straightforward than the explicit fluxes. Therefore, in our simulation we used the flux of the metal precursor and the C/M ratio of the precursors as the independent variables.

### Intrinsic parameters

Different from the extrinsic parameters, which are explicitly represented in the simulation, the intrinsic parameters are represented in the form of energies or energy barriers. These energy relations can be acquired from finely controlled experiments. However, such experimental efforts prove to be highly challenging, but the data are much more easily accessible from density functional theory (DFT) simulation coupled with the nudged elastic band (NEB) method^47–49^ with reasonable accuracy. The set of parameters to describe the epitaxial behavior of WSe_2_ on a graphitic substrate have been calculated and presented in our previous publication^40^. In addition, it is helpful to sweep one parameter out of the DFT-based dataset to investigate how individual parameters guide the growth, and navigate in the multi-dimensional parameter space towards the optimal growth condition.

### Site energy, adsorption energy and transition energy barriers

**Figure 2:**
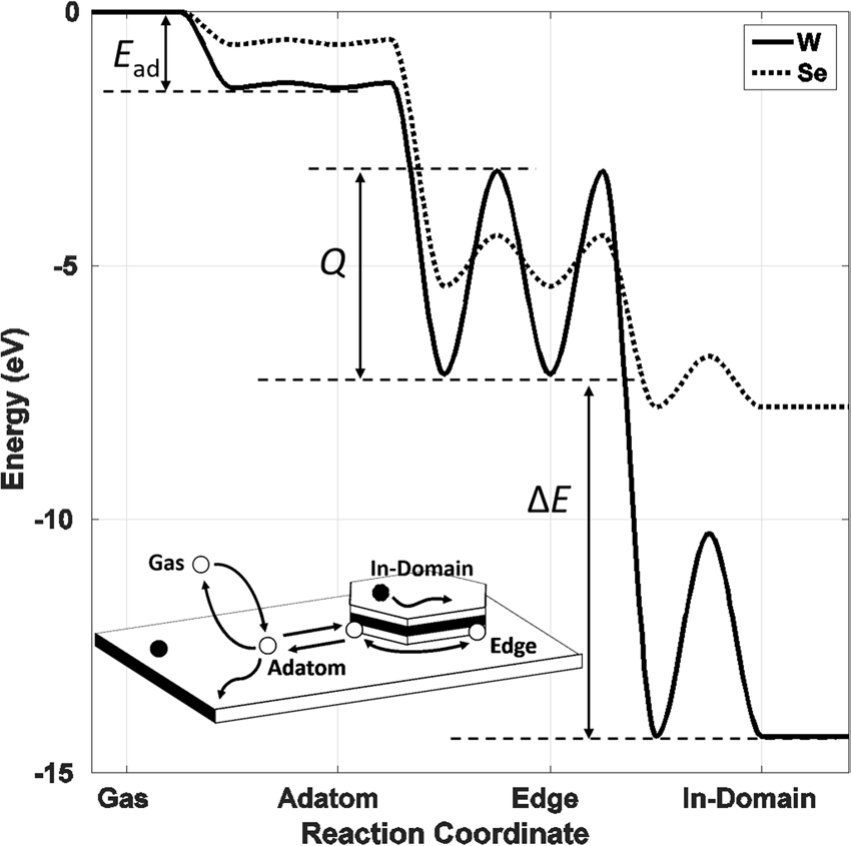
Simulated reaction energy diagram of the growth process. The simulation starts with adatoms; adatoms react with each other to form the TMD domains via free diffusion. After bonded into the domain, the in-flake atoms are mobile through edge diffusion and vacancy diffusion. Each state is defined by its state energy, calculated by the adsorption energy (*E*_*ad*_) and bond energy (*B*.*E*.). Each process is defined by its transition activation energy (*Q* or *Q* + Δ*E*). The energy diagram is based on the DFT calculations presented in a previous publication^40^.

In our model, the atoms can reside in three possible states: (1) atomic gas (not explicitly included), (2) freely diffusing adatoms on substrate, and (3) bonded atoms in TMD flake. The reaction scenario follows the sequence:

2 $$Gas\underset{des\mathrm{.}}{\overset{ads\mathrm{.}}{\rightleftharpoons }}\mathop{Adatoms}\limits_{\mathop{\circlearrowright }\limits_{diff\mathrm{.}}}\mathop{\rightleftharpoons }\limits^{diff\mathrm{.}}\mathop{Flake}\limits_{\mathop{\circlearrowright }\limits_{diff\mathrm{.}}}$$

and the reaction energy diagram is shown in Fig. 2.

Atomic sites are predetermined by the atomic arrangement (1T or 2H) of the TMD under study. Each site is defined by its energy, given by

3 $${E}_{site}={E}_{ad}+\sum _{neighboring}B\mathrm{.}E\mathrm{.},$$

in which *E*_*ad*_ is the adsorption energy, and is only applicable when the site is directly above the substrate or a full layer of TMD, *i*.*e*., in the (3n + 1)^*th*^ layer; the *B*.*E*. is the energy of bonds formed by the site and its first neighboring atoms. In this work, the states at 0 eV energy are set to be the empty atomic sites and the atoms in the gas phase. As Fig. 2 illustrates, in order to preserve the symmetry of a reaction, the transition between the two states is governed by the diffusion energy barrier and the energy difference between the two states, given by

4 $${r}_{A\to B}=\{\begin{array}{ll}{\nu }_{0}{e}^{-\frac{{Q}_{diff}}{{k}_{B}T}} & ,\,{\rm{\Delta }}{E}_{BA}\le 0\\ {\nu }_{0}{e}^{-\frac{{Q}_{diff}+{\rm{\Delta }}{E}_{BA}}{{k}_{B}T}} & ,\,{\rm{\Delta }}{E}_{BA}\, > \,0\end{array}$$

where *Q*_*diff*_ is the diffusion energy barrier between equivalent states, and Δ*E*_*AB*_ is the energy difference between the two states.

### Substrate

The growth of the TMDs on different substrates behaves very differently. This is the result of the different types of interactions between the reactants and the substrate. Graphitic substrates, such as highly oriented pyrolytical graphite, graphene, *etc*., form van der Waals gaps with the TMDs, and moderate chemical bonds with the adatoms. Metal oxides and metallic substrates, on the other hand, form strong chemical bonds with both the TMDs and the precursors. Important as it is, the role of the substrate has not been sufficiently addressed by previous simulation studies. In this work, the substrate effects are represented in the intrinsic parameters such as the adsorption energy of adatoms and the substrate diffusion energy barriers. With the substrate effects properly included, the vertical growth of the second layer TMD and above can be easily addressed by treating the underlying TMD layer as a different substrate for the 2^*nd*^ or higher layer growth.

## Growth rate and the conversion fraction

**Figure 3:**
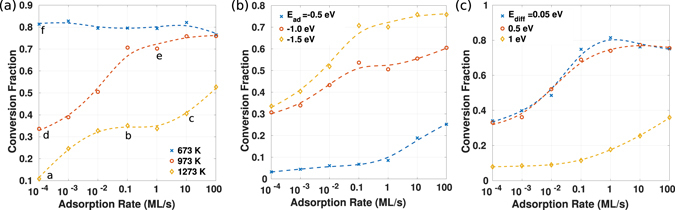
The conversion fraction, represented by the domains’ growth rate in monolayers per second divided by the adsorption rate (also in ML/s), as a function of the adsorption rate, under three different (**a**) substrate temperatures, (**b**) adsorption energies, and (**c**) substrate diffusion energy barriers. The resulting domain morphology under the conditions labeled in Fig. 3a are shown in Fig. 4.

In the study of the relation between the growth rate and the adsorption rate, the adsorption energy and substrate diffusion barrier for M and X are unified and changed together for the parametric study. The simulation shows that the growth rate increases in proportion to the increase of the adsorption rate, but the conversion of the adsorbed adatoms to the incorporated atoms into the domain is ineffective at elevated temperature. Figure 3a shows that while about 80% of adatoms participate in forming the TMD domain at 673 K, at an elevated temperature of 973 K, the conversion fraction is significantly lowered, due to the increased desorption rates. This means that at high temperature, the majority of the adatoms remains within the adsorption/desorption equilibrium cycles shown in Equation (2) rather than being converted into a domain. Adatom desorption and attachment are opposite processes influencing the conversion fraction, hence the growth rate. Adsorption energy dictates the desorption rate, as the desorption rate for an adatom is written as

5 $${r}_{des}={\nu }_{0}{e}^{-\frac{0-{E}_{ad}}{{k}_{B}T}}={\nu }_{0}{e}^{\frac{{E}_{ad}}{{k}_{B}T}}\mathrm{.}$$

The attachment rate, on the other hand, is determined by the density of adatoms, and their substrate diffusion energy barrier, as the adatoms are required to diffuse for a certain distance before they reach the domain edge.

The absolute value of the adsorption energy (1.5 eV) is much larger than the diffusion energy barrier (0.1 eV), and therefore at low temperature most adatoms are able to diffuse to the domain edge and attach before desorption. The Arrhenius relation (Equation (1)) indicates that as the temperature is increased, the fractional difference between two rates (determined by the transition state energy Δ*G* ^*≠*^) diminishes, making the two rates more comparable. Thus at high temperature, desorption, as the slower process, becomes comparable to the diffusion process thus leading to a reduced conversion fraction at high temperatures.

To further demonstrate that the conversion fraction is a result of the competition between the substrate diffusion and desorption, the adsorption energy and the diffusion energy barrier are varied separately, and the changes of the conversion fraction are recorded in Fig. 3b,c. When (the absolute value of) the adsorption energy is increased, desorption is suppressed and the conversion fraction goes up. When the substrate diffusion rate is reduced by a higher energy barrier, it becomes more difficult for the adatoms to reach a domain edge, resulting in a reduction in the conversion fraction.

Figure 3 also shows that increasing the adsorption rate increases the conversion yield, as it increases the chance of the adatom to find the domain edge by increasing the density of the adatoms.

## Domain morphology

Although a high adsorption rate coupled with a high conversion ratio results in high growth rate with better utilization of the precursors, our previous study has shown that a high growth rate always results in domains with a highly fractal morphology. The two morphologies (compact and fractal) and the transition between them have been observed in multiple systems^16, 21, 22, 50, 51^. The evolution of domain morphology with the variation of experiment conditions has been discussed in details in our previous publication^40^ and the highlights are recapitulated here.

**Figure 4:**
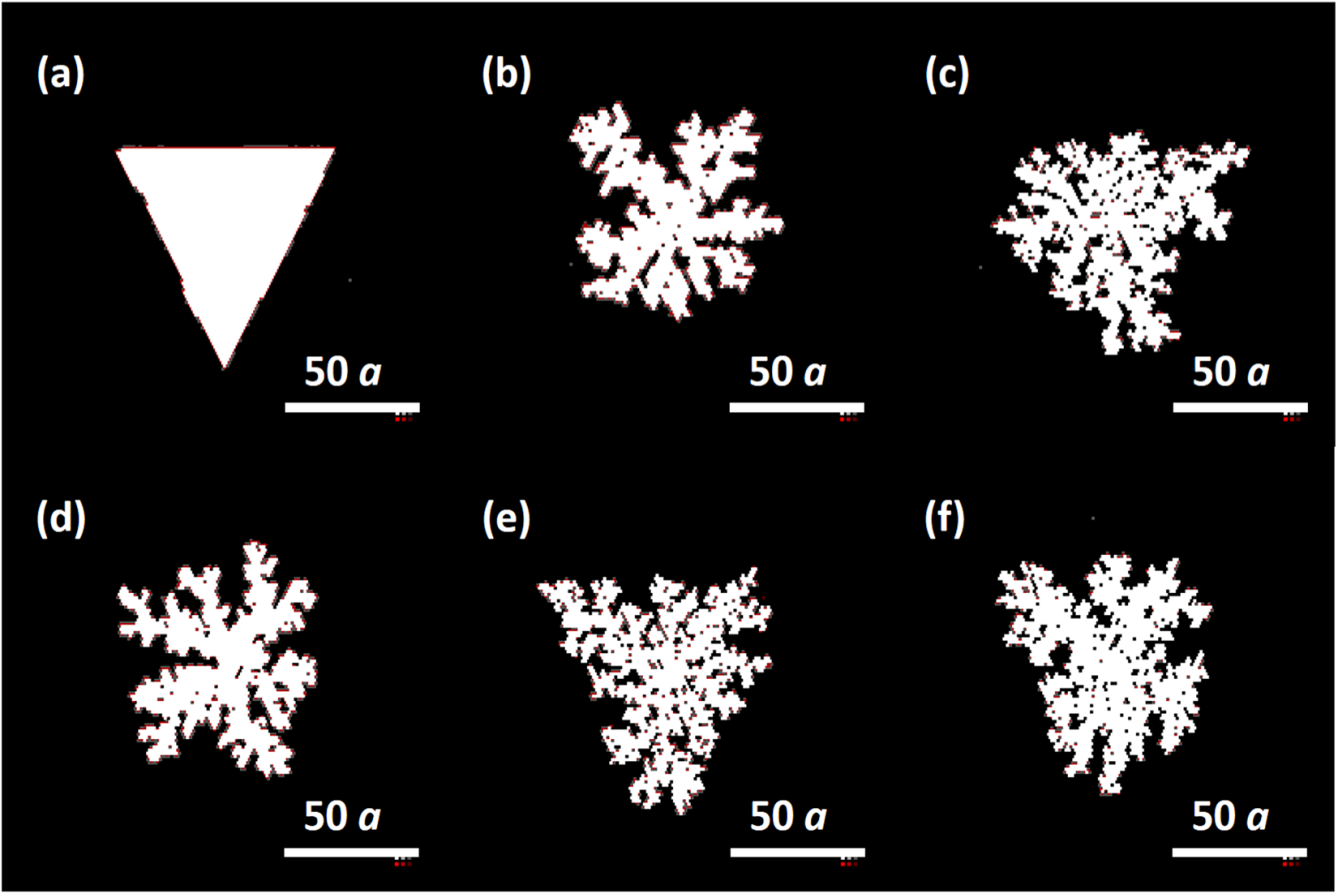
Domains’ morphology at different experimental conditions denoted in Fig. 3a. The scale bar is 50x lattice constant *a*. For WSe_2_, *a* = 332 pm, 50*a* = 16.6 nm.

Figure 4 shows the simulated domain shape under different conditions marked in Fig. 3a. Only when the conversion fraction is as low as below 10% (Fig. 4a), *i*.*e*., close to the adsorption/desorption equilibrium, can a compact triangular domain be generated. Otherwise the adsorption rate is either too low for attachment to happen, or so high that the domain begins to branch. A discontinuous structure is not desirable in device application because it can alter the electrical properties of the material considerably^52^. The fractal forming behavior is the result of a competition between atom attachment and the process of edge (or in-flake) diffusion, the latter being responsible for the relaxation of the domain from the initial shape (often random and branching) to the thermodynamically favorable configuration (compact triangle for the TMD monolayers). As the energy barrier for the in-flake diffusion is as high as 4.06 eV for W and 1.00 eV for Se, the relative difference between the rates of the in-flake and the substrate diffusion are well over four orders of magnitude, which is a rule of thumb limit of the transition from compact domains to the fractals^53, 54^. Based on the route shown in Equation (2), two ways are able to promote domain fractal shape, (1) the reduction of temperature, and (2) the increase on adsorption rate. Compact domains are produced close to the adsorption-desorption equilibrium, in which the conversion fraction and the growth rate are relatively low. This means that the film quality can be improved by lowering the growth rate. This improvement is prevalent regardless of the choice of the TMD or the substrate, as it is embedded in the general counteraction of addition and relaxation. In addition, post-deposition treatment such as annealing may be another way to improve the film quality after its initial growth^44^.

## Homogeneous Nucleation

**Figure 5:**
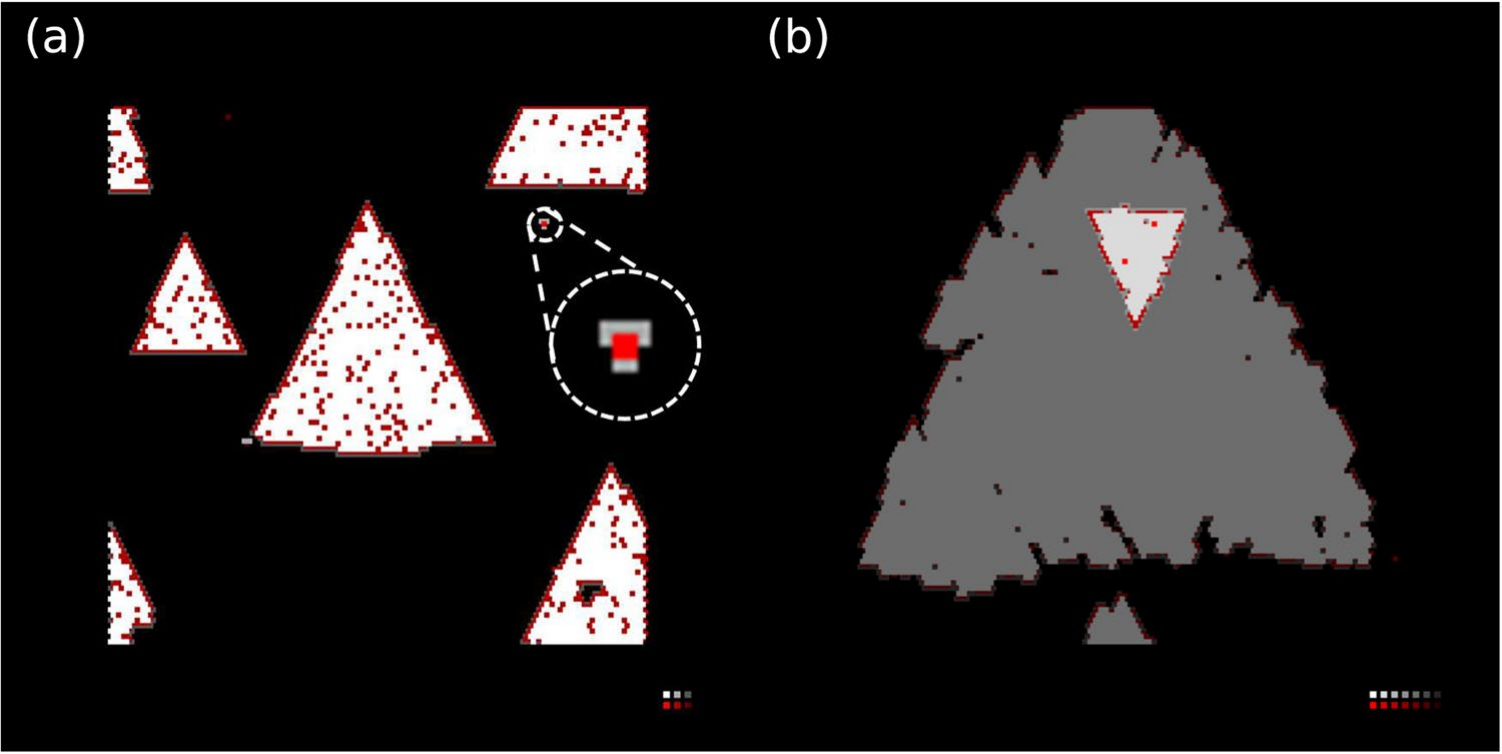
Examples of homogeneous nucleation on the substrate (**a**) and on another layer of TMD (**b**). The form of an initial nucleus (circled in (**a**) with a dashed circle) is zoomed in. Each pixel represents an atom color-coded by element (red: metal, gray: chalcogen) and the height is represented by brightness, as shown in the legend on the bottom right corner.

Nucleation density is of fundamental importance as it influences the resulting grain size of the TMD film which in turn affects many basic properties of the grown TMD materials. In the above simulations, a hexagonal nucleus was initially placed in the center of the substrate to study the growth of an existing nucleus. The heterogeneous nucleation scenario simulates the subsequent growth after the nucleation assisted by the imperfection of the substrate. In principle, they can be reduced as the substrate quality is improved. Nevertheless, spontaneous nucleation of additional domains on the substrate or the 1^*st*^ TMD layer was also observed at certain simulated growth conditions, without the assistance of any imperfections. This finding indicates that the KMC model in this work is capable of simulating the homogeneous nucleation, both on the substrate and on a TMD layer (Fig. 5). However, such nucleation is only sporadically observed during the investigation of the growth of the WSe_2_ on graphene, due to the difference on the condition promoting nucleation and that promoting a uniform domain growth. Different from the optimal growth condition, which requires higher temperature and lower adsorption rate to approach the adsorption/desorption equilibrium, homogeneous nucleation requires a further deviation from the equilibrium, in other word, a low temperature and a high adsorption rate.

**Figure 6:**
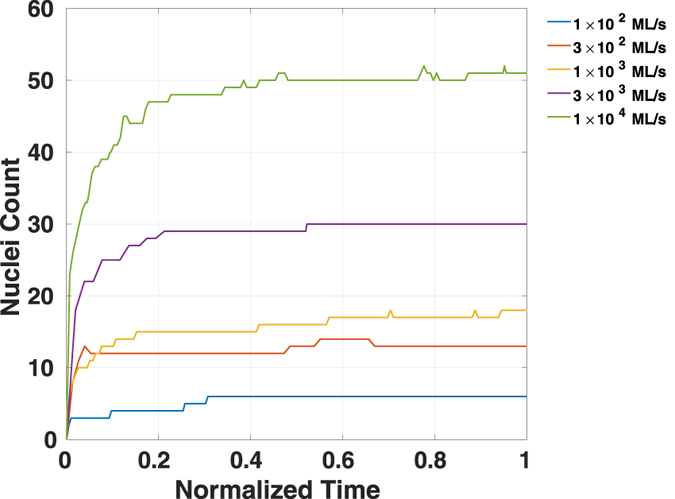
Homogeneous nuclei count as a function of normalized time under different adsorption rate at 773 K with the C/M ratio equal to 2.0. The nuclei count is within a substrate with the simulated area of 0.11 *μm* ^2^.

Figure 6 shows the evolution of the nuclei number as a function of time normalized by their respective time reaching a 10% coverage, under different adsorption rates. An increase of the nuclei number followed by a subsequent slowing down and saturation is observed. This finding indicates that the nucleation and growth processes are subject to a competition between nucleation and attachment events. The latter event has been identified earlier as a result of opposing adatom diffusion and desorption processes. In order for a nucleation event to happen, the adsorption population must win over desorption by a large margin; it is important to note that nucleation is a rare event as it calls for 3 to 4 atoms to arrive at the same spot via random Brownian diffusion (See the inset in Fig. 5a). Because it is easier for an adatom to be attached to an existing nucleus than to form a new one, additional nucleation will be statistically eliminated and saturation is observed when the nucleation density becomes comparable to that of the adatoms^54^.

Reduction of the saturated homogeneous nuclei concentration can be achieved by decreasing the density of the adatoms. According to the discussion in this work, the adatom population is subject to the complex influence of extrinsic parameters such as adsorption rate and temperature, and the intrinsic parameters such as the choice of the substrate.

## Lateral *vs*. vertical

In addition to controlling the grain size, it is also important to control the number of layers, as the electronic properties of the TMDs are very sensitive to the layer number. Thus the suppression of the vertical growth with respect to lateral growth is an important goal to achieve.

**Figure 7:**
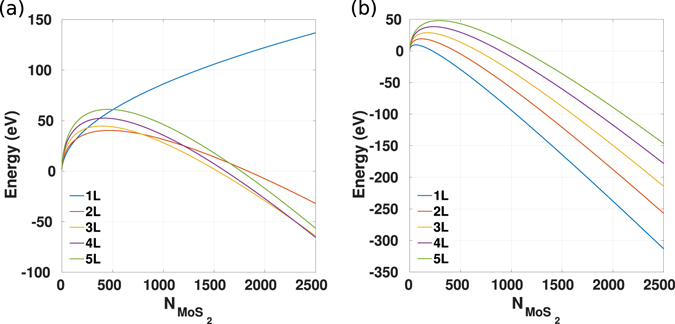
(**a**) The reproduction of Fig. 3(b) in ref. 55: the energy of isolated triangular flakes consisting of 1–5 layers (L) as a function of the number of MoS_2_ units. The curves intersect with each other. As a result, as the cluster size $({N}_{Mo{S}_{2}})$ increases, the layer number of the expected stable configuration also increases. Reproduced from the ref. 55 with permission from the Royal Society of Chemistry. (**b**) The result of the augmented model that includes the stabilizing effect of the substrate. As a result, the curves no longer intersect apart from the origin, and monolayer is always thermodynamically preferable with substrate.

It has been under debate whether the growth of monolayer TMDs can be achieved in principle, from a thermodynamic perspective. As the thermodynamic parameters for most of the bulk and monolayer TMDs are absent, trials have been made to use the density functional theory calculations to detect the theoretical limit of the layer number of the epitaxial flakes^55, 56^. Cuddy *et al*. have proposed an elegant analytic model to describe the formation of single and multi-layer TMD flakes, as a result of the competition between minimizing the formation of the destabilizing flake edges and maximizing the van der Waals stacks that stabilize the system:

6 $$E={N}_{L}3n\sigma +\frac{{N}_{Mo{S}_{2}}}{{N}_{L}}{E}_{vdW}({N}_{L}-1),$$

in which the first term represents the formation energy of the flake edges, and the second term represents the stabilizing energy of the van der Waals gaps. In Equation (6), *N*_*L*_ is the layer number, *n* is the edge length (in number of MoS_2_ units), *σ* is the edge formation energy (in eV/formula unit, 0.65 eV in the literature), ${N}_{Mo{S}_{2}}$ is the cluster size (in formula units), and *E*_*vdW*_ is the van der Waals interlayer energy (in eV per formula unit per van der Waals gap; *N*_*L*_ layers provide *N*_*L*_−1 van der Waals gaps; its value is −0.18 eV per formula unit in the literature). The case study showed a result as Fig. 7a ^55^. As the cluster size increases, the configuration containing more layer numbers becomes thermodynamically more favorable, as the generation of van der Waals stacks can make up the destabilizing effect of the domain edges.

The aforementioned model well justifies the thermodynamic origin of the domination of multilayered TMDs in isolation or in nature. However, when approaching the vdW epitaxy, the effect of the substrate must be taken into consideration, as it provides additional stabilization to the system. In order to take the substrate effect into account, Equation (6) can be expanded as:

7 $$E={N}_{L}3n\sigma +\frac{{N}_{Mo{S}_{2}}}{{N}_{L}}{E}_{vdW}({N}_{L}-1)+\frac{{N}_{Mo{S}_{2}}}{{N}_{L}}{E}_{sub},$$

in which *E*_*sub*_ is the substrate stabilizing energy (also in eV per formula unit), and the additional term represents the affinity between the substrate and the first layer flake. Even with a substrate that interacts with the grown flakes weakly, via van der Waals interaction that is comparable with the interlayer interaction (−0.18 eV per formula unit), the energy relation changes to Fig. 7b. These energy curves no longer intersect with each other away from the origin, and a supported monolayer is always thermodynamically favorable compared with its multilayer counterparts. This derivation shows that when approaching the growth of the layered materials, it is essential to include the effects of the substrate. This analysis also shows that judged by the thermodynamic criteria, the growth of monolayer TMD on substrate is preferred. Therefore, in order to understand the popular observations of multilayered growth of the TMDs, other factors such as the structural imperfection and kinetic issues must be included.

According to the conventional theories of the epitaxy of 3D materials, the most important kinetic factor for the competition between lateral and vertical growth is the Erhlich-Schwoebel (ES) barrier that hinders the step-down diffusion of the atoms landing on the terrace^57^. In these material systems, the desorption on the terrace is negligible and the extra ES barrier causes an accumulation of atoms on the terrace which promotes adatoms to nucleate and results in vertical growth. In vdW epitaxy, however, the binding of an adatom on a completed layer is not as strong. Therefore, the path of the removal of adatoms by desorption is introduced into the competition, and the nuance on the diffusion barrier is no longer significant enough to cause the atom accumulation. As a result, the growth of the second TMD layer and beyond follows the same nucleation-and-growth scenario as the first layer from their respective adatoms kinetics. The imbalance between the in-plane and the out-of-plane interactions will cause the ES barrier to diminish at the edge of a TMD domain. The fact that the atoms arrange themselves in the layered structure indicates that within each layer, the in-plane bonds have saturated the valence electrons of chalcogen atoms. Although an adatom can be chemically adsorbed on such a TMD layer, it has a strong preference to migrate to the domain edge and be incorporated into the domain. Such a strong preference has a reduced diffusion barrier at the flake edge, leading to a *negative* ES barrier, which is discovered in our DFT-NEB simulation (See the Supporting Information for details).

In the kinetic model presented in this work, the competition between the lateral growth and the vertical growth is in fact the competition between the substrate and the TMD itself as another substrate for additional layer growth. The proper inclusion of the substrate into the model in this work makes it possible to make the comparison. Our previous study has shown that for the case of WSe_2_ growth on the substrate of graphene, the adsorption energies of adatoms on WSe_2_ are slightly lower than those on graphene, but the diffusion energy barriers are nearly 4 times larger for Se and over 40 times larger for W^40^. Both these differences lead to a lower conversion fraction (and hence the growth rate, see Fig. 3) and the homogeneous nucleation density. In addition, from a statistical perspective, the probability of atoms’ arrival on top of the TMD surface versus that on the substrate is proportional to the area coverage of the 1^*st*^ layer domain. As a result, the vertical growth is seldom observed in the simulation of WSe_2_ on graphene at low coverage, which is also in agreement with the thermodynamic analysis. For a different TMD or a different substrate, a comparison can be made in a similar manner.

The comparison in this section is based on the assumption that the growth of the TMD layers is initiated by the homogeneous nucleation only. In reality, however, due to the difficulty of the fine control of the experimental conditions, and the imperfection of materials, heterogeneous nucleation and defect-assisted nucleation can dominate the experimental growth conditions. It is noteworthy that the current model serves as a theoretical limit of the experiments. In order to precisely simulate the cases closer to the experimental observations, more details are required in the model including the extrinsic defect nuclei.

## Discussion

It has been accepted by the research community that during an MBE growth, precursors only in the atomic form contribute to the reaction, due to the strong binding energy of the metal and chalcogen clusters^58^. The current model, as illustrated in Equation (2), agrees very well with such understanding of MBE growth processes. This KMC model has been used to guide the MBE synthesis of WSe_2_ with significantly improved film quality and controlled layer number, which will be presented in a separate publication. For a precise modeling of the MOCVD process where precursor molecules take part in the reactions, an expansion of the model from Equation (2) to:8is required. Equation (8) includes the chemical transition from the precursor in the molecular form to the actual atom, which is conceptually straightforward. However, in order to accurately capture the energetic and kinetic relations behind the chemical reactions, many more detailed theoretical simulations are necessary.

Equation (8) still limits the reaction at the surface of the substrate and the edge of the flakes. It is justifiable to exclude the gas phase reactions, as the high binding energy of the organic precursors makes it unlikely to dissociate and react in the gas phase^51^. In these cases, the domain edges act as the catalytic center that assists the breaking down of the precursor molecules. Further augmentation is required to simulate the synthesis methods involving the gas-phase reactions, such as CVT and the CVD methods using metal oxides and elemental chalcogens as the precursors.

## Conclusion

In this work, an efficient simulation method with rich atomic details is established to study the van der Waals epitaxy process of layered compound such as the TMDs. The simulation is capable of revealing the complex competitions that control different aspects of the growth including the substrate effects. Parameters that affect the growth rate, domain shape, homogeneous nucleation and layer number are discussed. With the appropriate expansion and addition of details, this simulation method can provide both qualitative and quantitative precision in comparison to experimental growth processes.

## Methods

The rejection-free kinetic Monte Carlo code is written in C language, following the introduction of the reference by Battaile^29^. The efficiency optimizations, particularly the binary search methods, follow the directions of the reference by Chatterjee and Vlachos^41^.

For the density functional theory calculation coupled with climb-image nudged elastic band method (DFT-CINEB), please refer to the Supporting Information.

## Supporting Information

The details and the results of the DFT-CINEB simulation on the ES barrier of WSe_2_ are presented in the Supporting Information.

## Electronic supplementary material

Supplementary Information
