# Supplementary material for: A kinetic Monte Carlo simulation method of van der Waals epitaxy for atomistic nucleation-growth processes of transition metal dichalcogenides
Source: Sci Rep. 2017 Jun 7;7:2977. doi: 10.1038/s41598-017-02919-2 (PMC5462835; doi:10.1038/s41598-017-02919-2)
Supplement: Supplementary file 1 — Supplementary Information [file 41598_2017_2919_MOESM1_ESM.pdf]

Supplementary Information: A kinetic Monte Carlo simulation method of van der Waals epitaxy for atomistic nucleation-growth processes of transition metal dichalcogenides

Yifan Nie[1], Chaoping Liang[1], Pil-Ryung Cha[2], Luigi Colombo[3], Robert M. Wallace[1], and Kyeongjae Cho[1,*]

[1]Department of Materials Science and Engineering, The University of Texas at Dallas, Richardson, Texas 75080, United States
[2]School of Advanced Materials, Kookmin University, Jeongneung-gil 77, Seongbuk-gu, Seoul, 136-702, Korea
[3]Texas Instruments Incorporated, 13121 TI Boulevard, Dallas, Texas 75243, United States
[*]kjcho@utdallas.edu

DFT-NEB Calculation of the Ehrlich-Schwoebel barriers of WSe_2

The density functional theory (DFT) is coupled with the climb-image nudged elastic band (CI-NEB) method[1–3] to study the downward diffusion of an W or Se adatom at a WSe_2 edge (Figure 1). The calculations are done using the Vienna Ab-initio Simulation Package (VASP) code[4]. The valence electronic states are expanded in a set of periodic plane waves and the interaction between ions and the valence electrons is implemented through the projector augmented wave (PAW) approach[5]. The Perdew-Burke-Ernzerhof (PBE) GGA exchange correlation functional is applied in the calculations[6]. The wave functions are expanded in plane waves with a kinetic energy cutoff of 400 eV and the convergence criteria for the electronic and ionic relaxation are 10^{-4} eV and 0.05 eV/Å, respectively. Integration over the Brillouin zone is performed with a Γ-centered $1 \times 2 \times 1$ k-point mesh. A supercell consisting of 4×3 rectangular unit cells of monolayer WSe_2 is used to simulate the domain edge. Vacuum layers of 20 Å is added to the *a* and *c* direction. During the simulation, rigid body assumption is employed which regards the atoms within the flake as immobile.

The NEB results shows an energy barrier of 0.17 eV for W and no barrier for Se. Comparing with their respective lateral diffusion barriers on WSe_2, both barriers are reduced (Table 1). For a 3D crystal, the Ehrlich-Schwoebel barrier is defined as the additional diffusion barrier encountered by a surface atom when crossing an atomic step. In the case of WSe_2, when adatoms cross the atomic step, they do not encounter an additional energy barrier, therefore there is no effective Ehrlich-Schwoebel barrier in the material system of WSe_2.

References

1. Jónsson, H., Mills, G. & Jacobsen, K. W. Nudged elastic band method for finding minimum energy paths of transitions. In Berne, B. J., Ciccotti, G. & Coker, D. F. (eds.) *Classical and Quantum Dynamics in Condensed Phase Simulations*, 385 (World Scientific, Singapore, 1998).

Table 1. Energy barriers of adatoms' lateral diffusion on a WSe_2 surface[7] and those of the adatoms' downward diffusion at a WSe_2 edge.

Diffusion barriers (eV)	Lateral	Downward
W	1.34	0.17
Se	0.23	0

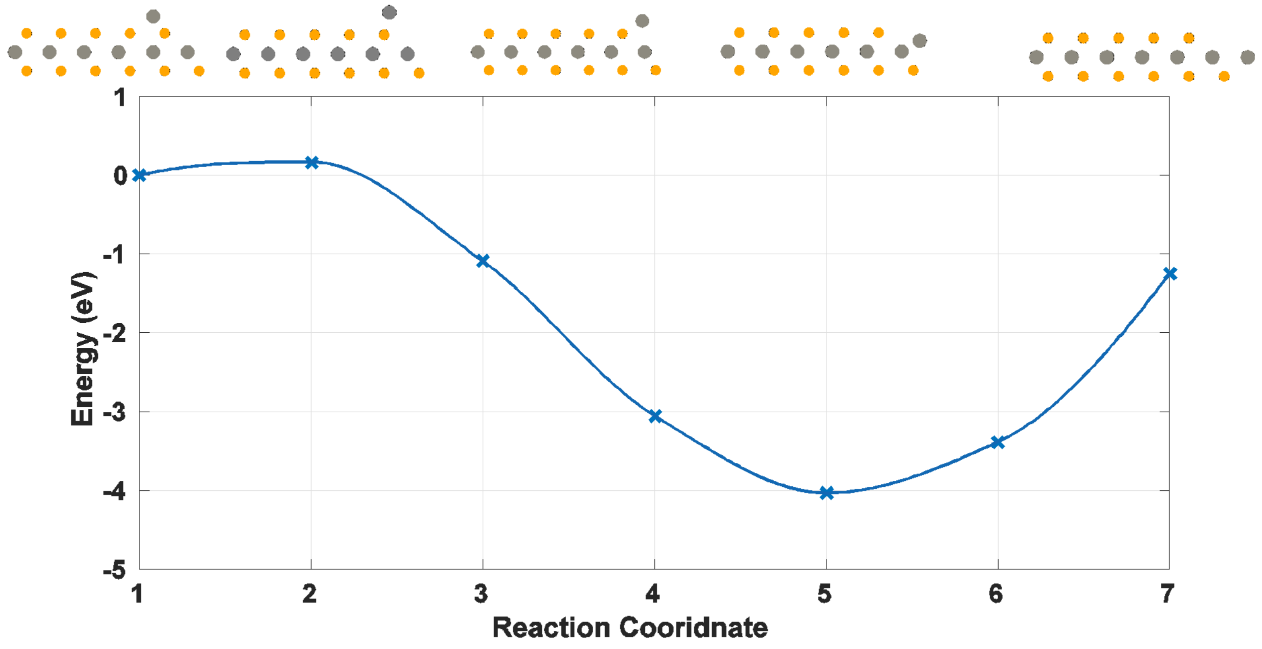

(a)

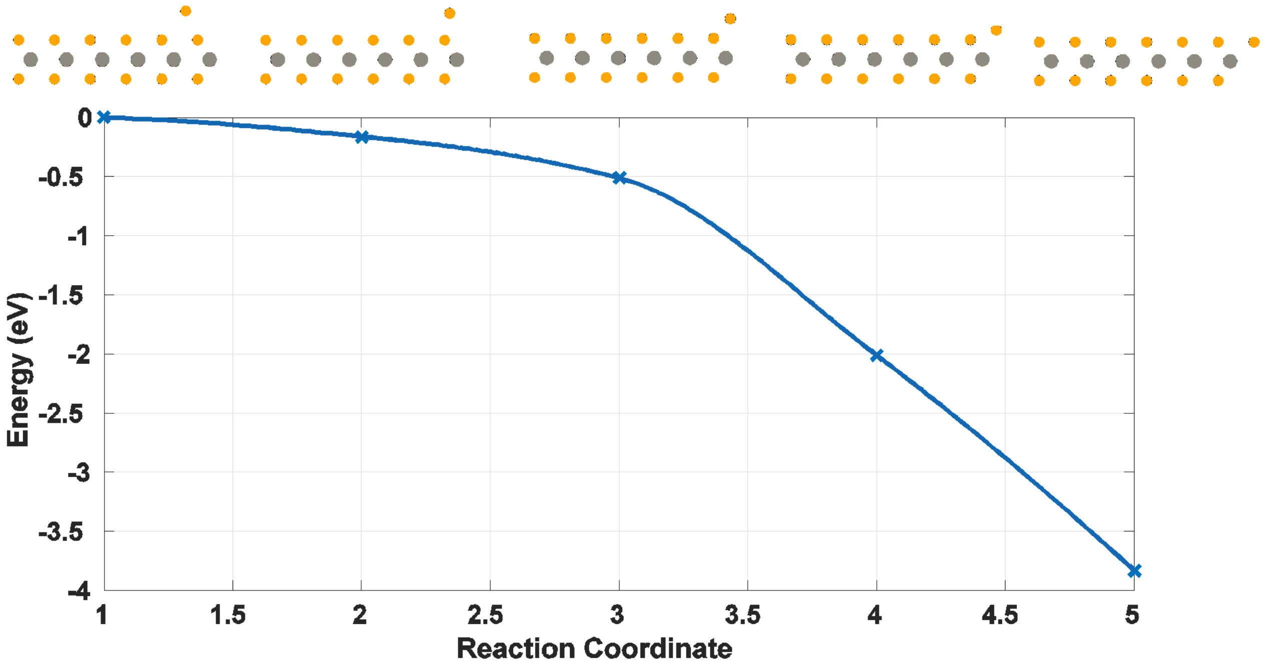

(b)

Figure 1. The diffusion paths of a W (a) and Se (b) atom on a WSe_2 domain edge from an adatom to the incorporation into the domain edge.

2. Henkelman, G., Uberuaga, B. P. & Jonsson, H. A climbing image nudged elastic band method for finding saddle points and minimum energy paths. *J. Chem. Phys.* **113**, 9901 – 9904 (2000).

3. Henkelman, G. & Jonsson, H. Improved tangent estimate in the nudged elastic band method for finding minimum energy paths and saddle points. *J. Chem. Phys.* **113**, 9978 – 9985 (2000).

4. Kresse, G. & Furthmüller, J. Efficient iterative schemes for *ab initio* total-energy calculations using a plane-wave basis set. *Phys. Rev. B* **54**, 11169–11186 (1996). URL http://link.aps.org/doi/10.1103/PhysRevB.54.11169. DOI 10.1103/PhysRevB.54.11169.

5. Kresse, G. & Joubert, D. From ultrasoft pseudopotentials to the projector augmented-wave method. *Phys. Rev. B* **59**, 1758–1775 (1999). URL http://link.aps.org/doi/10.1103/PhysRevB.59.1758. DOI 10.1103/PhysRevB.59.1758.

6. Perdew, J. P., Burke, K. & Ernzerhof, M. Generalized gradient approximation made simple. *Phys. Rev. Lett.* **77**, 3865–3868 (1996). URL http://link.aps.org/doi/10.1103/PhysRevLett.77.3865. DOI 10.1103/PhysRevLett.77.3865.

7. Nie, Y. *et al.* First principles kinetic monte carlo study on the growth patterns of wse 2 monolayer. *2D Mater.* **3**, 025029 (2016). URL http://stacks.iop.org/2053-1583/3/i=2/a=025029.
